# Supplementary material for: A novel approach of overtaking maneuvering using modified RG method
Source: PLoS One. 2022 Jan 20;17(1):e0260455. doi: 10.1371/journal.pone.0260455 (PMC8776333; doi:10.1371/journal.pone.0260455)
Supplement: Attachment — Submitted filename: Recommendation.docx [file pone.0260455.s005.docx]

1、The overtaking manoeuvre is a 2-dimensional movement. The literature review is poor. No relevant studies are introduced. At least, the following two references should be cited:

(1) Zhao, J., Knoop, V.L., Wang, M., 2020. Two-dimensional vehicular movement modelling at intersections based on optimal control. Transportation Research Part B: Methodological 138, 1-22.

(2) Bichiou, Y., Rakha, H.A., 2018. Developing an optimal intersection control system for automated connected vehicles. IEEE Trans. Intell. Transp. Syst. 20, 1908–1916.

2、RG technique is originally introduced for spaceships rendezvous missions with space stations and asteroids. Why is RG technology used in overtaking in this paper? What are the advantages of this method over traditional overtaking methods?

3、The scope of application of this method should be explained. leading vehicle may change speed, lane, etc. is this method applicable? In addition, the influence of lane changing behavior of rear vehicles on overtaking cannot be ignored.

4、The improved RG technology is better than that before the improvement. This conclusion is easy to draw. But will the improved RG model be better than the traditional classical overtaking models? The improved method should be compared with the classical overtaking models.
