# Supplementary material for: A novel approach of overtaking maneuvering using modified RG method
Source: PLoS One. 2022 Jan 20;17(1):e0260455. doi: 10.1371/journal.pone.0260455 (PMC8776333; doi:10.1371/journal.pone.0260455)
Supplement: Attachment — Submitted filename: Response to Reviewers.docx [file pone.0260455.s006.docx]

**Original Manuscript ID:** PONE-D-21-28089

**Original Article Title: “A Novel Approach of Overtaking Maneuvering using Modified RG Method”.**

**To:** Editor

**Re:** Response to reviewers

Dear Editor,

Thank you for allowing a resubmission of our manuscript, with an opportunity to address the editor’s and reviewers’ comments. We do hope that you will find the manuscript up to the mark. We are very much willing for the paper to be assigned to the same reviewers.

We have addressed all the comments of editor which are explained in detail in ‘response to editor section’. We are also uploading (a) our point-by-point response to the comments (below) (response to reviewers), (b) an updated manuscript with yellow highlighting indicating changes, and (c) a clean updated manuscript without highlights (PDF main document).

Best regards,

Usman Ghumman et al.

**Response to editor:**

**Editor’s concern # 1:**

Please ensure that your manuscript meets PLOS ONE's style requirements, including those for file naming. The PLOS ONE style templates can be found at 
<https://journals.plos.org/plosone/s/file?id=wjVg/PLOSOne_formatting_sample_main_body.pdf> and 
<https://journals.plos.org/plosone/s/file?id=ba62/PLOSOne_formatting_sample_title_authors_affiliations.pdf>

**Author’s response:**

Thank you for guiding us.

**Author’s action:** We have transformed our article according to the format given. Same font style and size is followed. Title page is also similar to the sample provided.

**Editor’s concern # 2:**

 In your Data Availability statement, you have not specified where the minimal data set underlying the results described in your manuscript can be found. PLOS defines a study's minimal data set as the underlying data used to reach the conclusions drawn in the manuscript and any additional data required to replicate the reported study findings in their entirety. All PLOS journals require that the minimal data set be made fully available. For more information about our data policy, please see <http://journals.plos.org/plosone/s/data-availability>.

**Author’s response:**

Thank you very much for suggesting this short coming. We respect your opinion. In our research article all data is available in tables.

**Author’s action:** Both reviewers have replied ‘Yes’ for the question asked regarding data availability which underlay the findings of result. In our humble opinion no action is required.

**Editor’s concern # 3:** We note that Figure 28 in your submission contain copyrighted images.

**Author’s response:**

Thank you for identification of copy righted image.

**Author’s action:** Fig 28 was a graphical abstract. It is removed from revised edition.

**Response to reviewers:**

**The response to Reviewer is highlighted in yellow color in the revised paper.**

**Reviewer # 1: (concern 1)**

The overtaking maneuver is a 2-dimensional movement. The literature review is poor. No relevant studies are introduced. At least, the following two references should be cited:
(1) Zhao, J., Knoop, V.L., Wang, M., 2020. Two-dimensional vehicular movement modelling at intersections based on optimal control. Transportation Research Part B: Methodological 138, 1-22.
(2) Bichiou, Y., Rakha, H.A., 2018. Developing an optimal intersection control system for automated connected vehicles. IEEE Trans. Intell. Transp. Syst. 20, 1908–1916

**Author’s response:** Thank you for supporting our article. We respect your kind opinion. We have added 13 latest journal and international conference articles and identified research gap. Most of papers are published in last 4-5 years.

**Author’s action:** following two portions are added to the revised article. Comparison with conventional method [38] is also performed.

. In [13], authors proposed a practical approach for driver assistance during overtaking by analyzing the acceleration and lane of next vehicle from opposite direction. Radar and video camera sensors are used in sensors fusion concept. But it failed when multiple vehicles have same acceleration and moving in different lane. In [14], follow the gap algorithm was proposed for vehicle overtaking during tense situations i.e., multiple vehicles in same line and having same speed. Results were compared with sin-X algorithm [15] and were found comparatively better. But follow the gap algorithm is not efficient when next vehicle varies speed regularly or changes lane due to heavy traffic density. X-sin function uses mathematical function for analyzing the scenario for comfortable overtaking maneuvering. It calculates longitude and lateral position of the ego vehicle. Technology independent sensor (TIS) is implemented via MATLAB and Pre-Scan software’s by [16], which scan geometries of the vehicles to assists driver in safe overtaking maneuvering. Acceleration and vehicle direction were not considered which reduced the accuracy. Hierarchical overtaking technique for driver assistance is proposed in [17] which uses acceleration and speed signals from the surrounding vehicles and uses them in clustering methods to achieve high probability density function, which results in predicting expected motion. Computed reference is then tracked using the Linear Parameter Varying (LPV) control design method to guarantee safe motion. This proposed technique is very complex and requires lot of computational power. It can handle high density traffic interactions. [18] proposed deep learning-based reinforcement framework method for generating vehicle motion model, but huge data set is required for training safe overtaking safe maneuvering strategy. In [19] trajectory optimization for safe overtaking is performed via behavior and trajectory planning algorithm. This technique minimizes intrusion onto the adjacent lane. The proposed technique is very slow in performance therefore it is not adopted for further implementation. But this technique is more accurate than all mentioned techniques. [20] proposed brix model for overtaking maneuvering of two tankers. Moment acting on the ship hulls and maneuvering motion were analyzed for safe overtaking. Special function was established for calculating minimum distance, but this technique is only valid for two vehicles. Behavior of 3010 vehicles were analyzed in [21] during overtaking in China. Nonparametric survival analysis was performed to model the overtaking time before conducting log-rank test. It was found that conventional vehicle is faster in overtaking than electrical vehicles, as well as men are also safer than women during overtaking. Dynamic trajectory planning algorithm is prosed in [22], in which overtaking maneuvering trajectory is divided into short time trajectories to manipulate safe overtaking. This method becomes complex for three lane road. In [23] overtaking maneuvering is taken as an optimization problem, pontryagain’s minimum principle is used to reduce the fuel consumption by 18% during overtaking period. However, constraints of the said scenario do not fit for every vehicle and high-density traffic. In [24] optimal and shortest path selection for overtaking maneuver is done using optimization algorithms, but this is only valid when a single vehicle has many choices for overtaking and have very low traffic density on road.

**Comparative analysis of RG algorithm with conventional off-line overtaking method:**

The comparison of the proposed on-line methodology was also carried with an off-line method presented in [38]. Since the technique proposed cannot cope with variations in obstacle velocity, for this comparison, both vehicles are moving with constant velocities. Two simulations are presented for this comparison: one with no obstacle is driving lane while another with no other vehicle present in driving lane. The results of the simulations for the comparison are presented via a set of two figures and one table. The first figure shows C’s path and velocity profile using the proposed modified RG method and the second figure shows C’s path and velocity profile using the off-line method. The table shows the time taken and the distance travelled using both methodologies.

**Case 1, Scenario 1**

There is no obstacle vehicle present which could delay/restrict the overtaking manoeuvre. In this scenario, vehicle to be overtaken, is moving with a constant velocity of 20 m/s and the velocity of C is 30 m/s. The results obtained from the simulation are presented in figures 22 and 23.

**Figure 22: Path and Velocity Profile of C Using the Modified RG Method**

**Figure 23: Path and Velocity Profile of C Using the Off-Line Method*.***

**Table 5. Basic Overtaking Parameters for Case 1, Scenario 1 – A Comparison**

|  | Modified RG Method | Off-Line Method |
| --- | --- | --- |
| Total time (s) | 13.5 | 15.5 |
| Distance travelled (m) | 430 | 466 |

**Case 2, Scenario 1**

There is no other vehicle is present which could delay/restrict the overtaking manoeuvre. In this scenario, both vehicles are moving with constant velocities of 20 m/s and 25 m/s, respectively. The starting velocity of C is taken as 30 m/s. The results obtained from the simulation are presented in Figure 24 and 25.

**Figure 24: Path and Velocity Profile of P Using the Modified RG Method**

**Figure 25:Path and Velocity Profile of P Using the Off-Line Method.**

**Table 6. Basic Overtaking Parameters for Case 2, Scenario 1 – A Comparison.**

|  | Modified RG Method | Off-Line Method |
| --- | --- | --- |
| Total time (s) | 34.2 | 39.5 |
| Distance travelled (m) | 844 | 966 |

**Reviewer # 1: (concern 2)**

RG technique is originally introduced for spaceships rendezvous missions with space stations and asteroids. Why is RG technology used in overtaking in this paper? What are the advantages of this method over traditional overtaking methods?

**Author’s response:** Thank you for pointing this. We honor your opinion. Explanation of this concern is added in revised edition in detail.

**Author’s action:** following text is modified accordingly.

RG technique is originally introduced for spaceships rendezvous missions with space stations and asteroids [31-32]. RG technique has delivered promising results for non-maneuvering targets interception which corresponds to vehicles moving in a straight path in our case. Therefore, this technique is adopted in our research article, in which six different scenarios are analyzed. However, there are two major limitations associated with RG technique. The first limitation is RG technique is designed for rendezvous missions with a particular target and it does not include overtaking the target. Secondly, numerous constraints are attached to chasing vehicle which are not associated with spaceships, so an amendment is required in technique to make it useful for optimal overtaking maneuver.

The first limitation is addressed by the introduction of a shadow target. Shadow target will guide ‘*C*’ through all the phases of overtaking to ensure user comfort and safety. The location of shadow target will be defined according to the location of vehicle which is to be overtaken. The second limitation is addressed by adopting RG technique proposed in [33-37] for robotic autonomous vehicle interception. The technique mentioned in [33-37] enabled us to gather relevant information of ‘*C*’, ‘*OB*’, and ‘*L*’ for generation of single acceleration command to be used for ‘*C*’. The acceleration command would enable ‘*C*’ to overtake ‘*OB*’ and simultaneously avoiding ‘*L*’ in a time-optimal manner. The acceleration command is calculated via velocity-matching with shadow target considering constraints applied due to chasing vehicle dynamics and user comfort. The shadow target will also avoid vehicle collision in overtaking lane by avoiding obstacle. When RG is compared to other overtaking techniques, it results in higher accuracy, less complexity and can model maximum scenarios.

**Reviewer # 1: (concern 3)**

The scope of application of this method should be explained. leading vehicle may change speed, lane, etc. is this method applicable? In addition, the influence of lane changing behavior of rear vehicles on overtaking cannot be ignored.

**Author’s response:** We are very much thankful to you for identifying the scenario. Leading vehicle may change speed as well as lane. In our research article minimum 2s time distance is maintained, therefore variation of speed and lane do not influence the proposed algorithm. Moreover, six different scenarios are modeled so that maximum possible situations can be handled.

**Author action:** Text is modified in article accordingly.

Leading vehicle changes speed and lane regularly, which is modeled in expected six scenarios. Initially, the scenario of an absence of any other vehicle in overtaking lane is considered. Once the distance between two vehicles gets 2s, RG algorithm creates imaginary target points *‘M_1_’, ‘M_2_’,* and *‘M_3_’* to ensure successful overtaking of leading vehicle ‘*L*’ by chaser vehicle ‘*C*’. The second scenario involves blocking vehicle ‘*OB*’ in overtaking lane which inhibits ‘*C*’ to overtake ‘*L*’. Therefore, proposed algorithm comes into effect and allows ‘*OB*’ to overtake ‘*L*’ first to create a room for ‘*C*’ to overtake ‘*L*’. The third scenario involves cancellation of overtaking decision after ‘*C*’ gets into overtaking lane. This situation arises when ‘*L*’ starts accelerating and the velocity of ‘*L*’ becomes greater than ‘*C*’ and eventually, ‘*C*’ needs to get back in driving lane. The final situation is complex in comparison with previous situations as it involves multiple vehicles ‘*L_1_*’ and ‘*L_2_*’ in driving lane and also the blocking vehicle ‘*OB*’ in overtaking lane. In this scenario, RG algorithm will not allow ‘*C’* to cross both vehicles straight away as it waits for a particular distance between ‘*OB*’ and ‘*L_1_*’- ‘*L_2_*’ to ensure the ‘*C*’ gets safely back into a driving lane after overtaking ‘*L_1_*’ and ‘*L_2_*’.

**Reviewer # 1: (concern 4)**

The improved RG technology is better than that before the improvement. This conclusion is easy to draw. But will the improved RG model be better than the traditional classical overtaking models? The improved method should be compared with the classical overtaking models.

**Author’s response:** We honor your suggestion. Improved method is further compared with classical offline method. Moreover, detailed literature review is done in revised edition in which research gap of each technique is mentioned. Improved RG method is better

**Author Action:** Following text regarding comparison with conventional offline method [38] is added in revised article.

**Comparative analysis of RG algorithm with conventional off-line overtaking method:**

The comparison of the proposed on-line methodology was also carried with an off-line method presented in [38]. Since the technique proposed cannot cope with variations in obstacle velocity, for this comparison, both vehicles are moving with constant velocities. Two simulations are presented for this comparison: one with no obstacle is driving lane while another with no other vehicle present in driving lane. The results of the simulations for the comparison are presented via a set of two figures and one table. The first figure shows C’s path and velocity profile using the proposed modified RG method and the second figure shows C’s path and velocity profile using the off-line method. The table shows the time taken and the distance travelled using both methodologies.

**Case 1, Scenario 1**

There is no obstacle vehicle present which could delay/restrict the overtaking manoeuvre. In this scenario, vehicle to be overtaken, is moving with a constant velocity of 20 m/s and the velocity of C is 30 m/s. The results obtained from the simulation are presented in figures 22 and 23.

**Figure 22: Path and Velocity Profile of C Using the Modified RG Method**

**Figure 23: Path and Velocity Profile of C Using the Off-Line Method*.***

**Table 5. Basic Overtaking Parameters for Case 1, Scenario 1 – A Comparison**

|  | Modified RG Method | Off-Line Method |
| --- | --- | --- |
| Total time (s) | 13.5 | 15.5 |
| Distance travelled (m) | 430 | 466 |

**Case 2, Scenario 1**

There is no other vehicle is present which could delay/restrict the overtaking manoeuvre. In this scenario, both vehicles are moving with constant velocities of 20 m/s and 25 m/s, respectively. The starting velocity of C is taken as 30 m/s. The results obtained from the simulation are presented in Figure 24 and 25.

**Figure 24: Path and Velocity Profile of P Using the Modified RG Method**

**Figure 25:Path and Velocity Profile of P Using the Off-Line Method.**

**Table 6. Basic Overtaking Parameters for Case 2, Scenario 1 – A Comparison.**

|  | Modified RG Method | Off-Line Method |
| --- | --- | --- |
| Total time (s) | 34.2 | 39.5 |
| Distance travelled (m) | 844 | 966 |

**Reviewer # 2: (concern 1)**

This paper focuses on the overtaking maneuvering for trajectory planning and obstacle avoidance. The topic is interesting, but the treatment of the methodology is weak. The authors should make great efforts to make this paper published.

**Author’s response:** Thank you for pointing this out. We have revised article. Revised article now consists of detailed literature review, comparison with conventional offline method, identification of research gaps of different techniques in literature review and revised six different overtaking scenarios.

**Author action:** Revised text is highlighted yellow in revised article.

**Reviewer # 2: (concern 2)**

The layout of this paper should obey the format.

**Author’s response:** Thank you very much for pointing this out. We honor your comment. Revised article is now according to the format given in authors section of the journal. Which are: <https://journals.plos.org/plosone/s/file?id=wjVg/PLOSOne_formatting_sample_main_body.pdf>  <https://journals.plos.org/plosone/s/file?id=ba62/PLOSOne_formatting_sample_title_authors_affiliations.pdf>

**Author action:** Revised article is now according to the format.

**Reviewer # 2: (concern 3)**

The figures are unclear and ambiguous for readers. And it is undesirable to include so many figures in this paper.

**Author’s response:** Thank you for identifying this. We respect your opinion. In our modest opinion, these figures helped us in explaining all six scenarios.

**Author action:** No action needed.

**Reviewer # 2: (concern 4)**

Many relative scientific papers are missing in the Introduction section.

**Author’s response:** We honor your suggestions. 13 latest articles are included in introduction section. Moreover, research gap of each article is also mentioned. Most of articles are published in previous 4-5 years.

**Author action:** introduction section is revised. Yellow highlighted portion is added.

In [13], authors proposed a practical approach for driver assistance during overtaking by analyzing the acceleration and lane of next vehicle from opposite direction. Radar and video camera sensors are used in sensors fusion concept. But it failed when multiple vehicles have same acceleration and moving in different lane. In [14], follow the gap algorithm was proposed for vehicle overtaking during tense situations i.e., multiple vehicles in same line and having same speed. Results were compared with sin-X algorithm [15] and were found comparatively better. But follow the gap algorithm is not efficient when next vehicle varies speed regularly or changes lane due to heavy traffic density. X-sin function uses mathematical function for analyzing the scenario for comfortable overtaking maneuvering. It calculates longitude and lateral position of the ego vehicle. Technology independent sensor (TIS) is implemented via MATLAB and Pre-Scan software’s by [16], which scan geometries of the vehicles to assists driver in safe overtaking maneuvering. Acceleration and vehicle direction were not considered which reduced the accuracy. Hierarchical overtaking technique for driver assistance is proposed in [17] which uses acceleration and speed signals from the surrounding vehicles and uses them in clustering methods to achieve high probability density function, which results in predicting expected motion. Computed reference is then tracked using the Linear Parameter Varying (LPV) control design method to guarantee safe motion. This proposed technique is very complex and requires lot of computational power. It can handle high density traffic interactions. [18] proposed deep learning-based reinforcement framework method for generating vehicle motion model, but huge data set is required for training safe overtaking safe maneuvering strategy. In [19] trajectory optimization for safe overtaking is performed via behavior and trajectory planning algorithm. This technique minimizes intrusion onto the adjacent lane. The proposed technique is very slow in performance therefore it is not adopted for further implementation. But this technique is more accurate than all mentioned techniques. [20] proposed brix model for overtaking maneuvering of two tankers. Moment acting on the ship hulls and maneuvering motion were analyzed for safe overtaking. Special function was established for calculating minimum distance, but this technique is only valid for two vehicles. Behavior of 3010 vehicles were analyzed in [21] during overtaking in China. Nonparametric survival analysis was performed to model the overtaking time before conducting log-rank test. It was found that conventional vehicle is faster in overtaking than electrical vehicles, as well as men are also safer than women during overtaking. Dynamic trajectory planning algorithm is prosed in [22], in which overtaking maneuvering trajectory is divided into short time trajectories to manipulate safe overtaking. This method becomes complex for three lane road. In [23] overtaking maneuvering is taken as an optimization problem, pontryagain’s minimum principle is used to reduce the fuel consumption by 18% during overtaking period. However, constraints of the said scenario do not fit for every vehicle and high-density traffic. In [24] optimal and shortest path selection for overtaking maneuver is done using optimization algorithms, but this is only valid when a single vehicle has many choices for overtaking and have very low traffic density on road.

**Reviewer # 2: (concern 5)**

The contribution of this paper is unclear.

**Author’s response:** Thank you very much for pointing this out. We honor your kind opinion. In our modest opinion, our paper does carry significant contribution in terms of novel approach of implementing modified RG algorithm for six different scenarios. First time six different scenarios are modeled in a single article.

**Author Response:** revised contribution of this paper is as:

The major contribution of the article are as follows.

1. Devising a methodology based upon RG technique that not only performs well by treating vehicle in driving lane as an obstacle but also yields effective results in presence of blocking vehicles in overtaking lane alongside leading vehicles in driving lane.
2. A novel modified form of RG technique is proposed and its comparative analysis with conventional RG technique is also conducted to validate the effectiveness of the proposed approach.
3. Modified RG technique is also compared with conventional offline technique.
4. Six different scenarios are modeled, which caters almost maximum possible situations.

The uniqueness of manuscript is portrayed by an attempt to address the scenario of overtaking which involves multiple vehicles (driving and overtaking lane) through RG technique and proposed modified form of RG technique. The proposed technique yields better results as it allows 10% less time for overtaking. In addition, the decisions in these complex overtaking scenarios have always been made considering the safety and comfort of chasing vehicle user.

**Reviewer # 2: (concern 6)**

The paper contains many typos and errors of units.

**Author’s response:** Thank you very much for pointing out.

**Author action:** Whole text of this article is thoroughly read, and maximum possible effort has been put to remove typos and errors of units.

**Reviewer # 2: (concern 7)**

The Rendezvous Guidance technique should be expressed clearly in section 2.2.

**Author’s response:** Thank you for kind suggestions. Section 2.2 is revised. Moreover, mathematical section is also explained in detailed in revised article.

**Author action:** section 2.2 is revised.

**Rendezvous Guidance Technique**

The optimal overtaking maneuver for chasing vehicle can be planned via Rendezvous Guidance (RG) technique. RG technique is originally introduced for spaceships rendezvous missions with space stations and asteroids [31-32]. RG technique has delivered promising results for non-maneuvering targets interception which corresponds to vehicles moving in a straight path in our case. Therefore, this technique is adopted in our research article, in which six different scenarios are analyzed. However, there are two major limitations associated with RG technique. The first limitation is RG technique is designed for rendezvous missions with a particular target and it does not include overtaking the target. Secondly, numerous constraints are attached to chasing vehicle which are not associated with spaceships, so an amendment is required in technique to make it useful for optimal overtaking maneuver.

The first limitation is addressed by the introduction of a shadow target. Shadow target will guide ‘*C*’ through all the phases of overtaking to ensure user comfort and safety. The location of shadow target will be defined according to the location of vehicle which is to be overtaken. The second limitation is addressed by adopting RG technique proposed in [33-37] for robotic autonomous vehicle interception. The technique mentioned in [33-37] enabled us to gather relevant information of ‘*C*’, ‘*OB*’, and ‘*L*’ for generation of single acceleration command to be used for ‘*C*’. The acceleration command would enable ‘*C*’ to overtake ‘*OB*’ and simultaneously avoiding ‘*L*’ in a time-optimal manner. The acceleration command is calculated via velocity-matching with shadow target considering constraints applied due to chasing vehicle dynamics and user comfort. The shadow target will also avoid vehicle collision in overtaking lane by avoiding obstacle. When RG is compared to other overtaking techniques, it results in higher accuracy, less complexity and can model maximum scenarios.

**Reviewer # 2: (concern 7)**

How does this algorithm distinguish the overtaking scenarios? Sometimes two or more scenarios may occur at the same time. For instance, there are multiple vehicles, but the subject vehicle achieves the unsuccessful overtaking. Will this algorithm handle this situation?

**Author’s response:** Thank you for identifying it. We honour your kind opinion. Six different scenarios are identified. Our algorithm distinguishes scenarios by the concept of 2s distance. Each scenario is explained theoretically and graphically. Unsuccessful overtaking is not an option for vehicle in our research approach. Not overtaking and sustaining its original position is option instead.

**Author action: Introduction section is modified accordingly.**

**Reviewer # 2: (concern 8)**

In figure 24, it seems the two vehicles collide with each other, which is unreasonable.

**Author’s response:** Thank you for pointing this out. In fig 24 (fig 27 in revised paper) vehicles do not collied. It depicts comparison between simulation and experimental results. In experimental scenario pursuer vehicle is delayed by 10%-time distance.

**Author action: NO action needed.**
