# Supplementary material for: A novel approach of overtaking maneuvering using modified RG method
Source: PLoS One. 2022 Jan 20;17(1):e0260455. doi: 10.1371/journal.pone.0260455 (PMC8776333; doi:10.1371/journal.pone.0260455)
Supplement: Attachment — Submitted filename: Response to Reviewers v1.docx [file pone.0260455.s007.docx]

**Original Manuscript ID:** PONE-D-21-28089R1

**Original Article Title: “A Novel Approach of Overtaking Maneuvering using Modified RG Method”.**

**To:** Editor

**Re:** Response to reviewers

Dear Editor,

Thank you for allowing a resubmission of our manuscript, with an opportunity to address the reviewers’ comments. We do hope that you will find the manuscript up to the mark. We are very much willing for the paper to be assigned to the same reviewers.

We have addressed all the comments of reviewers which are explained in detail in ‘response to reviewer section’. We are also uploading (a) our point-by-point response to the comments (below) (response to reviewers), (b) an updated manuscript with yellow highlighting indicating changes, and (c) a clean updated manuscript without highlights.

Best regards,

Usman Ghumman et al.

**Response to reviewers:**

**The response to Reviewer is highlighted in yellow color in the revised paper.**

**Reviewer # 1: (concern 1)**

A novel modified form of RG technique is proposed and its comparative analysis with conventional RG technique is also conducted to validate the effectiveness of the proposed approach. The proposed technique yields better results as it allows 10% less time for overtaking. Modified RG technique is also compared with conventional offline technique. The proposed technique is technically sound, and the data support the conclusions.

**Author’s response:** Thank you for supporting our article.

**Author’s action:** No action needed.

**Reviewer # 2: (concern 1)**

Although there are so many figures in the revised manuscript (which however are not shown), the presented figures convey little information, and the explanation of the figures are quite poor. For instance, where is the overtaking lane and OB in Figure 7 to Figure 10?

**Author’s response:** Thank you for pointing this. We honor your opinion. Figures are reduced to 17 from 31. Figures showing single step are removed and only figures showing all steps are left. Moreover, overtaking and traveling lanes are identified in description of concerned figures.

**Author’s action:** Figures 2, 3 and 4 are merged. Figure 6 shows steps of figure 5, so both are merged. Figures 18,19,20,21 and 22,23,24,25 are explained in tables as well, so all are removed. Figure 29 is also removed because scenario is well explained in the text.

The whole scenario is depicted in Fig. 1. Upper lane in figure 1 is overtaking lane while lower lane is travelling lane. Same is the case in all scenarios figure.

During overtaking, the velocity of ‘C’ remains constant within each particular phase. The pictorial representation of overtaking scenario 1 is given in Fig. 4. Upper lane is overtaking lane while lower lane is travelling lane.

**Reviewer # 2: (concern 2)**

I did not see 6 scenarios in this paper. There are actually 4 overtaking scenarios. Be careful when using "case", "scenario" and "situation".

**Author’s response:** We are very much thankful to you for identifying typo mistake. It is addressed.

**Author action: “**Six scenarios” is replaced with “four scenarios” in article accordingly.

1. Modified RG technique is also compared with conventional offline technique.
2. Four different scenarios are modeled, which caters almost maximum possible situations.

**Reviewer # 2: (concern 3)**

As to Figure 11, the y-distance path subfigure fails to show the time in second, so relationship between the speeds and the lateral position is unclear. The same holds for the other similar figures.

**Author’s response:** We honor your query. Both portions of the figure are to be read continuously at a time. i.e. till forth second y-axis distance and speed is zero, but after 5^th^ second it increases. At 7-8 second it reaches to its maximum values and then reduces at 9-10^th^ second, and so on.

**Author Action:** No action needed.

**Reviewer # 2: (concern 4)**

The comparison between the modified and conventional RG algorithms under scenario 3 and 4 should also be included.

**Author’s response:** Thank you for your kind suggestion. We honor your suggestion. Revised article now contains the comparison between the modified and conventional RG algorithms under scenario 3 and 4 as well.

**Author action:** comparison is added.

The comparative analysis required that simulations which were performed for modified RG algorithm should be repetitively performed for the conventional RG method. All four scenarios are utilized for comparison of both methods. Tabular representation of the parameter values of the total time taken for vehicle to perform overtaking maneuver and distance travelled during overtaking maneuvering are done for each scenario for readers to grasp which method performs better.

**Overtaking Scenario 3**

In this scenario, chasing vehicle wants to overtake leading vehicle but speed of leading vehicle is more than chasing vehicle so chasing vehicle comes down to lower lane without overtaking. Initially starting speed of chasing vehicle is 20 m/s while speed of vehicle to overtaken is 25 m/s. during overtaking speed of vehicle to be overtaken increases to 30 m/s. The parameter values for both methods are listed in Table 5. In this case, time taken for returning to travelling in modified RG method is less which means that chasing vehicle has to cover less distance to complete overtaking maneuvering.

**Table 5.  Comparison of Time and Distance in Scenario 3**

| **Parameters** | **Modified RG Method** | **Conventional RG Method** |
| --- | --- | --- |
| Total time (s) | 24 | 26 |
| Distance travelled (m) | 730 | 740 |

**Overtaking Scenario 4**

In this scenario, chasing vehicle needs to overtake two leading vehicle and there is also blocking vehicle obstructing the path of chasing vehicle in overtaking lane. Initial velocity of chasing vehicle is 25m/s while vehicles to overtaken are having velocity of 20m/s. The parameter values for both methods are listed in Table 6. In this case, time taken for overtaking maneuver in modified RG method is less which means that chasing vehicle has to cover less distance to complete overtaking maneuvering.

**Table 6.  Comparison of Time and Distance in Scenario 3**

| **Parameters** | **Modified RG Method** | **Conventional RG Method** |
| --- | --- | --- |
| Total time (s) | 22 | 25 |
| Distance travelled (m) | 690 | 725 |

**Reviewer # 2: (concern 5)**

More comparison cases with the off-line overtaking method are encouraged since scenario 1 is quite simple.

**Author’s response:** Thank you very much for suggesting this. We honor your comment. Revised article now contains comparison with off-line overtaking of all four scenarios.

**Author action:** Article is revised accordingly.

The comparison of the proposed on-line methodology was also carried with an off-line method presented in [38]. Since the technique proposed cannot cope with variations in obstacle velocity, for this comparison, both vehicles are moving with constant velocities. Simulations are performed for all four scenarios in this comparison. The results of the simulations for the comparison are presented via table. The table shows the time taken and the distance travelled using both methodologies.

**Scenario 1:**

There is no obstacle vehicle present which could delay/restrict the overtaking manoeuvre. In this scenario, vehicle to be overtaken, is moving with a constant velocity of 20 m/s and the velocity of C is 30 m/s. Table 7 shows basic overtaking parameters of both methods for scenario 1.

**Table 7. Basic Overtaking Parameters for Scenario 1 – A Comparison**

|  | Modified RG Method | Off-Line Method |
| --- | --- | --- |
| Total time (s) | 13.5 | 15.5 |
| Distance travelled (m) | 430 | 466 |

**Scenario 2**

There is another vehicle present in overtaking lane which could delay/restrict the overtaking manoeuvre. In this scenario, both vehicles are moving with constant velocities of 20 m/s and 25 m/s, respectively. The starting velocity of C is taken as 30 m/s. Table 8 shows basic overtaking parameters of both methods for scenario 2.

**Table 8. Basic Overtaking Parameters for Scenario 2 – A Comparison.**

|  | Modified RG Method | Off-Line Method |
| --- | --- | --- |
| Total time (s) | 34.2 | 39.5 |
| Distance travelled (m) | 844 | 966 |

**Scenario 3**

Chasing vehicle is having slow speed as compared to vehicle to be overtaking, so overtaking does not happen. In this scenario, chasing vehicle and vehicle to be overtaken are moving with initial velocities of 25 m/s and 20 m/s, respectively. Velocity of vehicle to be overtaken increases to 30m/s, so the chasing vehicle comes back to travelling lane. Table 9 shows basic overtaking parameters of both methods for scenario 2.

**Table 9. Basic Overtaking Parameters for Scenario 3 – A Comparison.**

|  | Modified RG Method | Off-Line Method |
| --- | --- | --- |
| Total time (s) | 23 | 27 |
| Distance travelled (m) | 720 | 770 |

**Scenario 4**

Chasing vehicle is to overtake vehicle with double lanes and in the presence of obstructing vehicle in overtaking lane. Table 10 shows basic overtaking parameters of both methods for scenario 2.

**Table 10. Basic Overtaking Parameters for Scenario 4 – A Comparison.**

|  | Modified RG Method | Off-Line Method |
| --- | --- | --- |
| Total time (s) | 22 | 25 |
| Distance travelled (m) | 690 | 725 |

**Reviewer # 2: (concern 6)**

Why the indicator values of the modified RG method in Table 3, 5 and 6 are different? The simulation settings when comparing with the conventional RG and the off-line overtaking method are supposed to be identical.

**Author’s response:** We honor your query. Indicator values of modified RG method in table 3,5 and 6 are different due to different initial velocities of chasing and vehicle to overtaken. In case of table 3, initial velocities are 18 m/s and 22m/s for chasing and vehicle to be overtaken respectively. While in case of table 5, initial velocities are 20m/s and 25m/s for chasing and vehicle to be overtaken respectively. And in table 6 these are 25m/s and 30m/s for chasing and vehicle to be overtaken respectively. So, both parameters, time and distance travel vary with respect to initial velocity.

**Author action:** No action needed.

**Reviewer # 2: (concern 7)**

There are still many grammar and typo errors throughout the paper. Please check.

**Author’s response:** Thank you for pointing this out. We honor your kind suggestions.

**Author action:** Manuscript is checked thoroughly and revised. Maximum possible effort has been done in this regard.
